# A novel framework for discovery and reuse of typical process route driven by symbolic entropy and intelligent optimisation algorithm

**DOI:** 10.1371/journal.pone.0274532

**Published:** 2022-09-12

**Authors:** Chunlei Li

**Affiliations:** 1 School of mechanical engineering, Baoji University of Arts and Sciences, Baoji Shaanxi, P.R. China; 2 Shaanxi Key Laboratory of Advanced Manufacturing and Evaluation of Robot Key Components, Baoji Shaanxi, P.R. China; Sant Longowal Institute of Engineering and Technology, INDIA

## Abstract

Manufacturing enterprises accumulate numerous manufacturing instances as they run and develop. Being able to excavate and apply the instance resources reasonably is one of the most effective approaches to improve manufacturing and support innovation. A novel framework for the discovery and reuse of typical process routes driven by symbolic entropy and intelligent optimisation algorithm so as to scientifically determine reuse objects and raise the reuse flexibility is proposed in this paper. A similarity measurement method of machining process routes based on symbolic entropy is developed in this framework. Subsequently, a typical process route discovery method based on the ant colony clustering model and similarity measurement is devised, and two reuse approaches based on the typical process route are analysed. Finally, three case studies are rendered. These case studies cover the aspects of similarity analysis, mining, and reuse of manufacturing instances, which systematically explains the whole procedure of discovery and reuse based on typical process route. The case studies show that (i) the similarity measurement method based on symbolic entropy can accurately evaluate the similarity among ten machining process routes, (ii) ant colony clustering model can realize adaptive clustering for these ten process routes, and (iii) indirect reuse approach for the typical process route can support the generation of new machining plan effectively. This reveal that the proposed framework comprehensively considers various aspects of retrieval and reuse of manufacturing instances, which can effectively support process instance reuse. Can better support process instance reuse.

## 1. Introduction

As manufacturing enterprises run and develop, they accumulate many manufacturing instances. These instances stand for the important data and knowledge resources and reflect the manufacturing capacity and level of the enterprises, which are a key embodiment of the design preferences and experience of the technologists in these enterprises. The ability to reasonably excavate and apply the instance resources can effectively improve manufacturing and support innovation [1]. Meanwhile, mining and application of existing instance resources is a focus of current and emerging research in the knowledge engineering field, which can lay a good foundation for transforming and upgrading manufacturing enterprises and realising intelligent manufacturing.

Determining the most valuable and potentially reusable objects is the main problem to be solved in order to discover and reuse high-value processing schemes. Among the numerous process schemes, some schemes exhibit typicality and representativeness. The processing routes of these typical schemes can be named typical process routes. They can be considered standard process routes extracted from those parts having similar structural features and functions [2]. Compared with other process routes, typical process routes contain rich process design rules and experience pertaining to specific parts. For example, in case of a simple ladder axis, it is composed of multi-segment cylindrical surfaces. Cylindrical surfaces can be obtained by turning, and their dimensional precision will be assured by finish cutting and grinding. So the typical process route of step shaft parts may be described as follows: rough turning → semi-finish turning → finish turning → grinding. Compared with the typical process route, although the shaft parts that are used in engineering can be more complicated, there will be no significant difference for main processing procedure, which reveals the typical process route has rich process rules and higher reuse value. Selecting a typical process route as the high-value object and achieving discovery and reuse of the typical process route have considerable value and significance; this can provide better support for machining process planning.

To discover the typical process route, the following two important issues must be addressed to satisfy the mining requirement of the route: similarity measurement and cluster analysis of machining process routes. The research on similarity measurement of manufacturing processes is usually the core issue and key technology in this field and has produced many significant results. Kambhampati [3] focused on selecting the reusable process from more alternative planning schemes and developed a method to calculate the distance between the alternative planning schemes and target objects. Liu et al. [4, 5] established a mathematical model of machining process routes, used the Manharstein distance to evaluate the similarity of two processing operations, and solved the similarity calculation problem of process routes by applying the Euclidean distance. Zhang et al. [6] coded processing operations according to the machinery industry standards, extracted the longest similar subsequence of two process sequences to construct the measurement factor, and established a multilevel similarity calculation formula of machining process routes. Zhou et al. [7] built a calculating model of process sequence similarity by analysing and comparing part features, including machining features and topological relations between features. They established a fuzzy similarity matrix of all the process sequences. Wang et al. [8] used the attributed directed graph to model the machining process. They measured the similarity between processes with the similarity between process cells and the similarity between process routes by the process model. Fan et al. [9] defined the similarity between processes cells and the similarity between process routes and then used self-adaptive affinity propagation clustering to discover typical processes. Peng et al. [10] considered that different product modules may have an identical or similar process route and mining the process similarity of product modules can effectively reduce production cost and improve production efficiency. They developed a novel product data mining approach with an improved Euclidean distance formula to analyse the product module process similarity. Wu et al. [11] proposed an innovative method based on process constituent elements model to objectively calculate the similarity of product manufacturing processes; the similarity model is established on the basis of six dimensions of process constituent elements, namely, input, output, resource, environment, value-added processing activity, and quality control and inspection. Among the aforementioned studies, the local similarity of consistent links between manufacturing processes is the main basis for similarity evaluation. Unfortunately, the impact of global similarity caused by the position of consistent links in manufacturing processes cannot be sufficiently considered. In addition to similarity measurement of machining process routes, cluster analysis of machining process routes is an important aspect of determining the typical process route. Clustering analysis is to mine some natural clusters from the data object collection to allow the data objects among clusters to be of higher similarity. The data objects in different clusters are of relatively small similarity. The clustering analysis method can be adopted to obtain the potential classification among data objects. In addition, based on this, the characteristics of data on each cluster can be observed and analysed. It is of important application value in fields such as knowledge mining, image processing, pattern recognition, medical diagnosis, bioengineering, and document retrieval. The clustering analysis methods can mainly be divided into traditional algorithm and modern clustering model based on swarm intelligence algorithm. Among traditional algorithms, the most extensively applied one is the K-means algorithm [12, 13]. However, one of its notable shortcomings is that the number of clusters must be initially specified. Furthermore, the initial position for the elements to be clustered can impose a significant impact on the clustering partition effect. The density-based clustering analysis methods are also one of the important research directions in traditional algorithms. In general, such methods map the data objects to the data space where the data class would be merged and divided based on distribution density. The advantages are the adaptability of clustering analysis problems distributed in any shape and the capability to eliminate the interference of the clustering process caused by the isolated data objects. However, the parameters of the algorithm are difficult to set. The relatively mature algorithms include DBSCAN [14], OPTICS [15], and DENCLUE [16, 17]. In recent years, artificial intelligence (AI) technology has received widespread attention for its application in the data mining field. Many scholars have started researching the clustering analysis model based on the swarm intelligence algorithm, for example, the ant colony optimisation (ACO) clustering algorithm [18, 19], clustering algorithm based on particle swarm optimisation (PSO) [20], artificial fish swarm clustering algorithm [21], bird swarm optimisation clustering algorithm [22], and moth swarm clustering algorithm [23]. Generally, compare with those classical clustering methods like K-means algorithm, the clustering analysis methods based on swarm intelligence algorithm are not affected by the setting of initial clustering conditions. In addition, owing to groupisation, such methods often exhibit rapid convergence rates and good global searchability. Therefore, based on the comprehensive consideration of algorithm maturity and universality, the ACO algorithm has been selected to construct the intelligent clustering model of the machining process route.

For reuse of the machining process route, the current mainstream approach is case-based reasoning (CBR) [24, 25]. Chang et al. [26] considered that retrieving a relevant case of process planning similar to the given part is an effective approach to develop a machining process plan. They adapted the retrieved case using CBR to generate the new process plan. Jiang et al. [27] presented a hybrid method combing rough set and CBR for remanufacturing process planning. A rough set was employed for feature reduction and rapid determination of features’ weights automatically, and CBR was utilised to calculate the similarity of process cases to identify the most suitable solution effectively from the case database. Li et al. [28] presented a hybrid method of blockchain and CBR for remanufacturing process planning. They utilised a blockchain network to record the remanufacturing knowledge and its associated transactions to guarantee the security and reliability of knowledge sharing. Further, they employed CBR to retrieve and reuse the most suitable solution by analysing the similarity between previous remanufacturing cases and a new case with the nearest neighbour algorithm. In these CBR methods, although the typical process route can be regarded as the reuse object to generate new processing schemes by reasoning, substantial human–computer interaction and revision are repeatedly required, which cause decreased flexibility and intelligence in reusing the manufacture instances.

A novel framework for discovering and reusing a typical process route driven by symbolic entropy and intelligent optimisation algorithm was developed in this study. A similarity measurement method of machining process routes based on symbolic entropy was developed, thus realising comprehensive consideration of global and local similarity of machining process routes. Subsequently, an intelligent clustering model based on ant colony algorithm and similarity measurement of machining process routes was built, classifying machining process routes and further extracting the typical process route in each class cluster. Finally, two reuse approaches based on typical process routes, including direct revision reuse and indirect matching reuse––this expands the reuse approach and improves reuse flexibility––were examined. Because the proposed framework comprehensively considers the aspects of similarity analysis, mining, and reuse of manufacturing instances, it can effectively support process instance reuse.

## 2. Discovery and reuse framework of the typical process route

The previous process plans can be considered as references for the technical process design of the new object so as to improve the design quality and efficiency. Meanwhile, in most cases, the difference of the majority of the series parts lies in the local structure and key dimension, but their machining procedures are extremely approximate. The flexible reutilisation of similar process plans can directly impact the research and development efficiency of the company’s products. To achieve the high reutilisation of the process plan, the insufficiency of orderly management for process cases must be addressed and a method that can divide the process route classification based on the similarity of process must be created. Thus, the effective classification and management of process plans can be achieved. Moreover, in each category, the typical process plan representing its category can be proactively analysed and extracted for reuse in the future. The typical process route represented in its category can be extracted for reference during the design of product process plans. For instance, the typical process route can serve as the reference example to contribute to new product development. Furthermore, the CBR technique can be applied to generating new process plans. In addition, the typical process can be served as the bridge for retrieval and reuse. The technologists can proceed with the similarity retrieval from all typical process routes to identify the most similar one to the design object. Subsequently, the secondary retrieval can be carried out from the above result to discover the most identical one to the reuse demand. By doing so, it is not necessary to retrieve all process plans. The reuse demand can be accurately matched while reducing the retrieval time.

**Fig 1:**
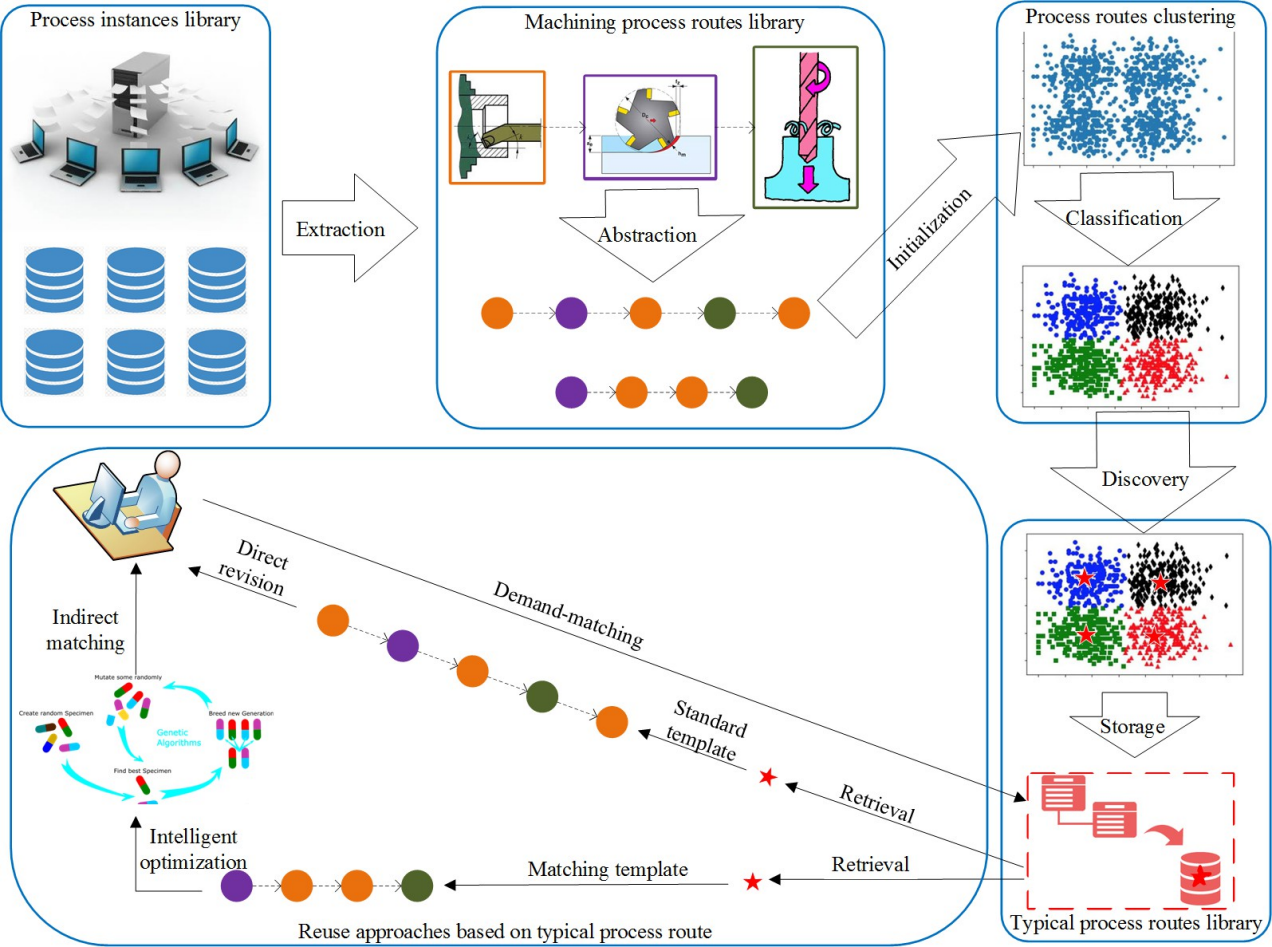
A framework for discovery and reuse of typical process route.

The discovery and reuse framework of the typical process route is shown in Fig 1. The previously accumulated process plans of the company are considered as the data objects to be clustered, and the similarity calculation results of various process routes serve as the reference for the clustering partition, retrieval, and reuse. Thus, the difficulty in reuse caused by the lack of rational organisation and management of previously accumulated process plans can be improved.

According to the afore described analysis, the discovery and reuse of typical process route mainly involve three modules, namely (1) the similarity calculation of machining process routes; (2) the clustering analysis of process routes based on the similarity calculation results and the acquisition of the typical process routes; and (3) the process reuse approaches based on the typical process routes.

## 3. Similarity measurement of machining process routes based on symbolic entropy

The modern machining processes of complicated products often involve multiple procedures, generally carried out at different workstations or equipment. The process route of the product reflects the sequence of machining processes, which contains the required types of manufacturing resources, their corresponding sequences, and the flow path at multiple stations (equipment) for the workpiece. The more similar the process route of two parts, the more similar would be the required types, quantity, sequence of manufacturing resources, and flow path of workpiece at the processing site; consequently, the higher would be the consistency of process plans.

If process routes are abstracted into processing sequences, the similarity measurement of machining process routes also can be transformed into similarity calculation of sequences. For the similarity calculation of sequences, the local and global information all should be considered. For two sequences, their longest common subsequence can be used to evaluate the global similarity, and the positions and frequency of their common elements can reflect the the local similarity. For instance, there are three character sequences as follows: acbea, abedd and aabef. The longest common subsequence is abe, which reflect the global similarity among them is close. In addition, the positions and frequency of common elements between character sequences acbea and aabef are same, so their similarity value is higher from the local information. In order to synthetically evaluate the local similarity and global similarity among process routes, a sequence similarity measurement method based on symbolic entropy has been adopted to evaluate the similarity among process routes. Firstly, the longest common subsequence should be extracted from the two process routes to be compared. Subsequently, each process method can be abstracted as a symbol to calculate the Shannon entropy of each position symbol in the longest common subsequence. The entropy difference among all positions can be utilised to compose the evaluation results of the subsequence similarity. As the calculation results of symbolic entropy are relative to the position and the frequency of occurrence of the symbol, the ultimate subsequence similarity calculation results obtained can effectively reflect the global and local information, thus strengthening the reliability of the calculation results. Finally, the similarity evaluation results of the longest common subsequence and the length of two original process routes to be compared should be comprehensively considered to obtain a similarity calculation result that can be relatively accurate and reliable and can integrate local, global, and length information.

### 3.1 Acquisition of longest common subsequence

The acquisition of the longest common subsequence is essentially a problem to locate the longest subsequence from two sequences to be compared. The longest common subsequence is defined as follows: if a sequence *S* = {*s*_1_,*s*_2_,⋯,*s*_*r*_} (integer *r* denotes the length of sequence *S*) is simultaneously a subsequence to two sequences *M* = {*m*_1_,*m*_2_,⋯,*m*_*u*_} (integer *u* denotes the length of sequence *M*) and *N* = {*n*_1_,*n*_2_,⋯,*n*_*v*_} (integer *v* denotes the length of sequence *N*) to be compared, that is, if Eq (1) is satisfied, *S* is considered the longest common subsequence of *M* and *N* if and only if the length *r* of sequence *S* takes the maximum value.


$$\left\{ \begin{array}{l} s_{x}=m_{y}=n_{z},\\ 1\leq y\leq u,\\ 1\leq z\leq v, \end{array}.\right.$$ (1)

The acquisition of the longest common subsequence is the basis of the application of bioinformatics, which is also a relatively classical problem requiring computer dynamic programming solutions. The existing literature [29–32] and tools (such as the Diff tool) have afforded relatively mature and universal solutions to such questions, which are not reiterated herein.

### 3.2 Calculation of symbolic entropy

The Shannon entropy was adopted in this study to calculate the symbolic entropy of each position procedure in the process routes. In sequence *M*, the symbol may appear many times in the same procedure; the position information for the *i*^th^ occurrence of some procedure symbol and its occurrence density can be described as *α*_*i*_, as follows:

$$\alpha_{i}=\frac{1}{p_{i}-p_{i-1}},\;1\leq i\leq k.$$ (2)

Here, *p*_*i*_ refers to the position of the *i*^th^ occurrence for the procedure symbol; *k* denotes the frequency of occurrence for such symbol in the sequence. Evidently, *p*_0_ can be considered the position of the procedure symbol before it appears. Let *p*_0_ = 0. Clearly, the position information for the *i*^th^ occurrence of some procedure symbol can be directly expressed by the value of *p*_*i*_, and its occurrence density can be described by used the difference value between *p*_*i*_ and *p*_*i*-1_. If the previous occurrence of the procedure symbol is fixed that *p*_*i*-1_ is a definite value, a higher value of *p*_*i*_ shows that (i) the *i*^th^ occurred position of some procedure symbol is later in the sequence, and (ii) the interval to its previous occurrence is larger. According to this feature, Eq (2) can be built. As known from Eq (2), the later the position of the occurrence of the procedure symbol in the sequence and the larger the interval to the previous occurrence, the smaller would be the value of *α*_*i*_. As a result, *α*_*i*_ can be adopted to describe the position of the *i*^th^ occurrence of the procedure symbol and the intensive interval to the previous occurrence of the same symbol.

To describe the orderliness of *α*_*i*_, the discrete probability distribution function expressed as Eq (3) can be established.


$$Q_{i}=\frac{\sum_{j=1}^{i}\alpha_{j}}{\sum_{j=1}^{k}\alpha_{j}},\;1\leq i,j\leq k.$$ (3)

The Shannon entropy of the procedure symbol on each position in the process route can be further calculated as

$$H(P_{t})=-Q_{i}\log_{2}Q_{i},\;1\leq i\leq k,1\leq t\leq r.$$ (4)

Here, *P*_*t*_ signifies the position of the *t*^th^ procedure symbol in *M*; *H*(*P*_*t*_) refers to the symbolic entropy of procedure at the position of *P*_*t*_; *P*_*t*_ is the *i*^th^ occurrence in *M*; and *Q*_*i*_ stands for its distribution probability.

### 3.3 Sequence similarity calculation based on symbolic entropy

In terms of any two process routes *M* = {*m*_1_,*m*_2_,⋯,*m*_*u*_}、 *N* = {*n*_1_,*n*_2_,⋯,*n*_*v*_} to be compared, the longest common subsequence *S* = {*s*_1_,*s*_2_,⋯,*s*_*r*_} can be extracted. If the position of some procedure symbol *s*_*x*_ in *S* is *P*_*y*_, and the position in *N* is *P*_*z*_, the similarity of both sequences can be calculated according to Eq (5).

$$sim(M,N)=1-\frac{1}{r}\sum_{x=1}^{r}\left|H(P_{y})-H(P_{z})\right|.$$ (5)

Here, *H*(*P*_*y*_) denotes the calculation result of Shannon entropy for procedure symbol *s*_*x*_ in *M*; *H*(*P*_*z*_) refers to the calculation result of Shannon entropy for procedure symbol *s*_*x*_ in *N*. The similarity calculation results of two process routes are composed of the Shannon entropy difference of all symbols in the longest common subsequence. Apparently, if the occurred position of the longest common subsequence in two original sequences and the interval between the occurrence of the same symbol are identical, the similarity obtained from the calculation would be high. Moreover, the symbolic entropy also reflects the global and local structure information of the original sequence, avoiding the scarcity of consideration of global structure information due to the dependence on the subsequence measurement. As a result, the accuracy of similarity calculation is improved.

In practice, if the lengths of two sequences to be compared are considerably different, it is impossible to determine whether they are of higher similarity even if the occurred position and frequency for the longest common subsequence in the original sequence is relatively approximate. For instance, in case of character sequences ‘aabedf’ and ‘aabe’, the longest common subsequence is aabe. The occurred position and frequency interval for aabe in the two original sequences are identical; however, this information cannot simply determine that these two sequences are completely identical. The impact caused by the inconsistent sequence length should be considered. The correction factor that can reflect the inconsistent length should be introduced to establish the final similarity formula, as follows:

$$SIM(M,N)=\frac{r}{\max(u,v)}sim(M,N).$$ (6)

The following simple example can describe the similarity measurement algorithm of the process route based on symbolic entropy. Assuming that there are two process routes: M (milling → grinding →boring → turning → drilling → boring) and N (milling → grinding →milling → boring → drilling). The longest common subsequence *S* (milling → grinding →boring → drilling) of them can be extracted. Then, the symbolic entropy in *M* and *N* for each machining element in *S* can be calculated according to Eqs (2)–(4), which are shown in Table 1.

**Table 1:** Calculation example of symbolic entropy.

*x*	1	2	3	4
*H*(*P*_*y*_)	0	0	0.5	0
*H*(*P*_*z*_)	0.39	0	0	0

Substitute the data in Table 1 into Eqs (5) and (6) to obtain the results of the similarity measurement of the machining sequence, which is as follows: $SIM(M,N)=\frac{4}{6}[1-\frac{1}{4}(\left|0-0.39|+|0.5-0\right|)]\approx 0.5183.$

## 4. Clustering analysis model of process routes and acquisition of their typical process

Clustering analysis is to mine some natural clusters from the data object collection to allow the data objects among clusters to be of higher similarity. The data objects in different clusters are of relatively small similarity. The clustering analysis method can be adopted to obtain the potential classification among data objects. In addition, based on this, the characteristics of data on each cluster can be observed and analysed. It is of significant application value in knowledge mining, image processing, pattern recognition, medical diagnosis, bioengineering and document retrieval. Considering the aspects of algorithm maturity and universality, the ACO algorithm was selected to construct the intelligent clustering model of the machining process route in this study.

### 4.1 Swarm similarity calculation of process route

In the ant colony clustering model of process route built in this study, all the routes are first placed in a two-dimensional (2D) plane randomly. In the event that an ant needs to pick up some process route under the non-loaded condition, the criterion depends on the comprehensive similarity between the process route and the other process route at its location, that is, swarm similarity. Similarly, if an ant needs to drop some process route under loaded conditions, the judgement standard relies on the swarm similarity among the current location where the ant is and all the other process routes.

On the basis of the process route similarity measurement method based on symbolic entropy mentioned in Section 3, the swarm similarity formula of process route can be expressed as

$$CSIM(M)=\frac{1}{|loca(M)|-1}\sum_{N\in loca(M),M\neq N}SIM(M,N).$$ (7)

Here, *CSIM*(*M*) stands for the swarm similarity of process route *M; loca*(*M*) refers to the location of *M* in the 2D grid plane; |*loca*(*M*)| signifies the quantity of process routes at location *loca*(*M*). If the non-loaded ant moves to *loca*(*M*) and determines whether to pick up *M*, |*loca*(*M*)|−1 would be equal to the quantity of all process routes at the location except for *M*; however, in the event that the loaded ant moves to *loca*(*M*) and determines whether to drop *M*, as *M* has moved to the current location along with ant, |*loca*(*M*)|−1 would then be equal to the quantity of all process routes at the location of the ant. It can provide a reference for the ant to determine whether to pick up or drop the process route by calculating the mean similarity for *M* and all other process routes at its location.

### 4.2 Process route clustering analysis based on ant swarm algorithm

**Fig 2:**
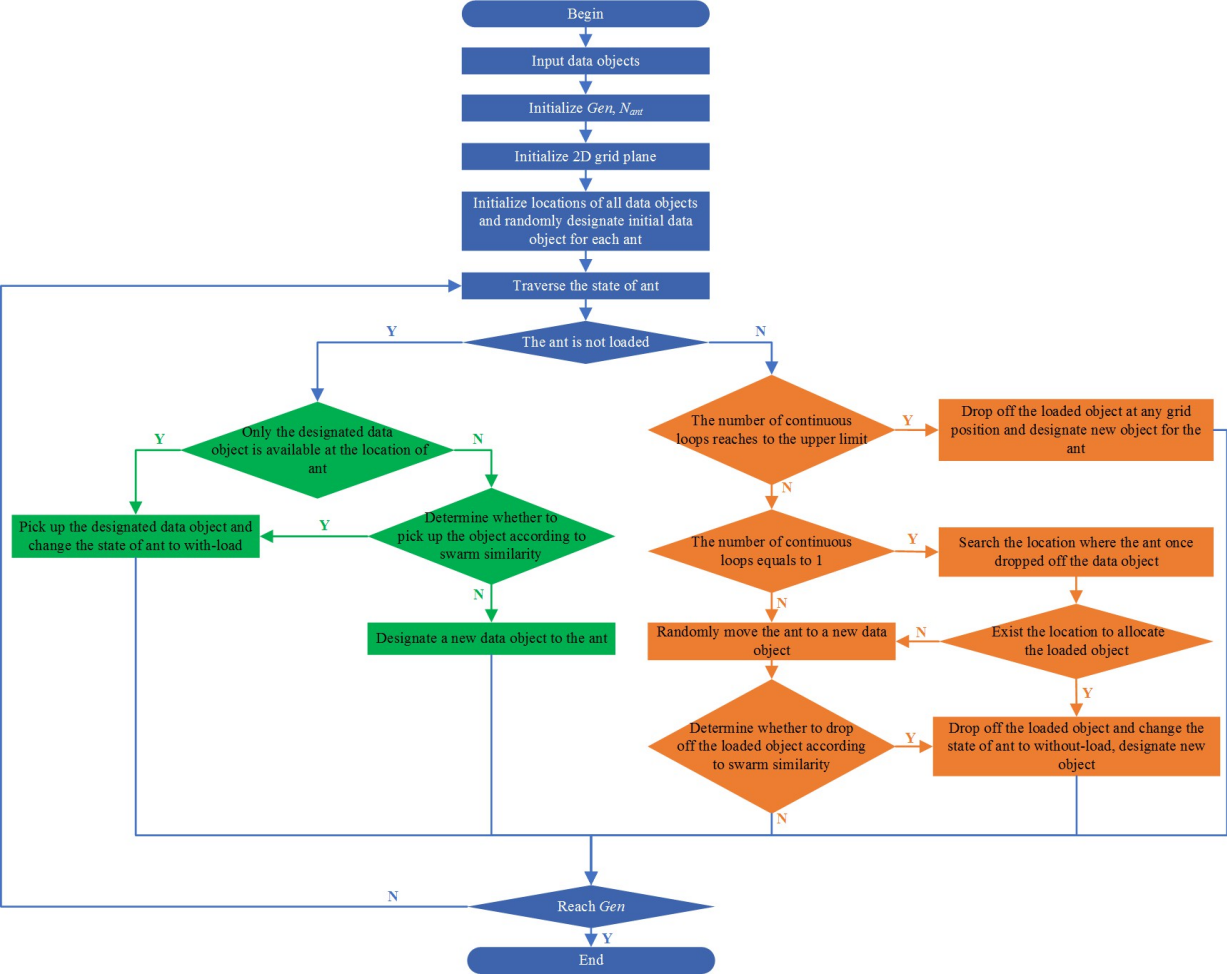
Flow chart of cluster analysis based on ant colony algorithm.

The clustering analysis flow of process route based on ant swarm algorithm is illustrated in Fig 2; the specific implementation steps are as follows:

Step 1: Initialise the iteration number *Gen* and the number of ant *N*_*ant*_. Here, *Gen* is generally determined by the size of the data object set. The more the process routes to be conducted the clustering analysis, the larger would be the *Gen*; the default value of *N*_*ant*_ is normally *N*_*ant*_ = *Num*/5, where *Num* refers to the number of process routes in the clustering analysis.

Step 2: Initialise the 2D grid plane. The size of the grid plan should adapt to the value of *Num* so as to ensure that the grid plane can accommodate all process routes without overlapping. In practice, the process routes are initially randomly distributed on the grid plane. Therefore, a slightly larger size should be selected so that two or more process routes would not randomly distribute to one location. The value range of abscissa *X* and ordinate *Y* on the 2D grid plane can be preset as $X=Y=3\sqrt{Num}$.

Step 3: The process routes to be clustered are randomly distributed to the grid plane defined in step 2. One process route *M* to be clustered can be considered as an example. For a positive integer *M*_*x*_ randomly generated from the range [1, *X*] and a positive integer *M*_*y*_ randomly generated from the range [1, *Y*], the location coordinates (*M*_*x*_, *M*_*y*_) are the initial random location of *M*.

Step 4: One process route should be randomly designated to each ant, and the initial location of the ant should be the one on the designated process route.

Step 5: Proceed with the clustering iterative loop.

Step 6: All ants should be traversed for each clustering loop. If some ant has no load, proceed with step 7; otherwise, move to step 8.

Step 7: The number of process routes at the ant’s location should be calculated. If there are no other process routes except for the designated process route *M*, that is, |*loca*(*M*)|−1 = 0, it indicates that this location is not the clustering location. As a result, *M* can be directly picked up, and the ant condition should turn into with-load. If other process routes are available except for *M*, Eq (7) should be utilised to calculate the swarm similarity among the designated process route and other routes at the location. In addition, the calculation result would help determine whether the ant should pick up the designated process route. There are two types of strategies to determine whether to pick up the designated object according to swarm similarity, as follows: (1) In the clustering analysis of small-scale data, the swarm similarity can be directly adopted as the judgement standard. Specifically, a threshold can be preset. When the swarm similarity is smaller than the preset threshold, the designated object can be picked up; the condition of ant should switch to be with-load; otherwise, a new object would be randomly designated to the ant and move the ant to the location of the new designated data object. (2) In the clustering analysis of large-scale data, increasingly many data object elements would enter each cluster, potentially reducing the similarity level of any individual to the entire swarm. As a result, the swarm similarity can be turned into pick-up probability to determine pick-up action. Specifically, the swarm similarity can be turned into pick-up probability *P*_*pick*_ according to space conversion probability formula [33], and *P*_*pick*_ can then be compared with random probability *P*_*ran*_. When *P*_*pick*_ ≥ *P*_*ran*_, the ant can pick up the designated object and switch to with-load condition; otherwise, a new object would be randomly designated to the ant, which would also be moved to the location of the new designated object.

Step 8: To improve the efficiency of the clustering algorithm, in terms of the ant that loads the process route, the considered priority should place the loaded process route to the location where the ant once dropped off the process route. The underlying rationale here is that the location where the ant used to drop off the data object is the potential clustering location. The blindness to conduct a direct global search can be avoided by judging such locations can be able to place the loaded data objects. If all locations where the ant used to drop off objects fail to satisfy the requirements to place down the loaded objects, the ant and its loaded data objects can be moved to a location of a new data object. The swarm similarity result can be utilised to determine whether the ant can place down the loaded process route at the current location. The reference to determine whether the ant can place down the loaded data object is also the value of swarm similarity, which specifically involves the following two strategies: (1) In the clustering analysis of small-scale data, the swarm similarity can be directly adopted as the judgement standard; specifically, a threshold can be preset. When such swarm similarity is smaller than the preset threshold, the designated object can be placed down––the condition of ant changes to non-load. Meanwhile, a new object should be designated to the ant. Accordingly, the ant should be moved to the location of the new designated data object. Otherwise, the ant should not drop off the loaded data object. (2) In the clustering analysis of large-scale data, increasingly many data object elements would enter each cluster with the clustering process, potentially reducing the similarity level of any individual to the entire swarm. As a result, the swarm similarity can be turned into drop-off probability to determine drop-off action. Specifically speaking, the swarm similarity can be turned into drop-off probability *P*_*drop*_ according to space conversion probability formula, and then compare *P*_*drop*_ with random probability *P*_*ran*_. When *P*_*drop*_ ≥*P*_*ran*_, the ant can drop off the designated object and switch to a non-load condition; simultaneously, a new object would be randomly formulated to the ant. Otherwise, the ant should not drop off the loaded data object. In addition to the aforedescribed strategies, the number of the iterative loop for the ant to load the same object should be regulated as each cluster may only contain one element, that is, when the iterative loop of ant reaches to a limit and fails to drop off the loaded data object, a randomly generated location on the 2D grid plane can be selected to drop off the loaded object. Meanwhile, a new data object should be designated to the ant.

Step 9: Check whether the number of iterative loops has reached *Gen*. If so, the loop should stop, and the clustering results should be output; otherwise, return to step 6 to continue the loop.

It can be seen from the above steps that the swarm intelligence clustering algorithm of process route proposed in this paper contains one big loop and one nested small loop. The former stands for the clustering iteration number. The latter represents that each clustering loop could iterate through all ants and completes the corresponding calculation and judgment. For each ant, the calculation and judgment of nested small loop has two selectable strategies, and their time-consuming is different which is hard to describe exactly. Here the time complexity of the proposed intelligent clustering algorithm can used O(*Gen*×*N*_ant_) to describe generally. Meanwhile, except for the pre-set parameters and defined data spaces, the temporary parameters mainly include that each ant may needs two variables that store *P*_*pick*_/*P*_*drop*_ and *P*_*ran*_ when it is performing one of two selectable strategies, so the space complexity can used O(*Gen*×*N*_ant_×2) to describe generally. Compared with other clustering algorithms, the swarm intelligence clustering algorithm of the process route proposed herein has four main advantages in terms of application.

First, the number of clustering loops and quantity of ant can be reasonably set according to the size of the data object set. On the one hand, it can ensure that each ant can traverse most of the objects and lower the missing detection rate; on the other hand, it can urge each data object can be examined by multiple ants many times, reducing the lapse.

Second, the number of objects at the location where the ant is determined before deciding whether to pick up the designated objects. The designated object would be directly picked up if it is not the clustering location. Otherwise, the swarm similarity would be calculated to determine whether to pick up the designated data object. Thus, the computational expense of the system can be reduced.

Third, two strategies are proposed to determine the pick-up and drop-off of the data object for the ant, which suits both large-scale and small-scale data clustering analyses, thus significantly improving the algorithm’s adaptability.

Finally, the algorithm sets up a storage space that records where an object is placed for each ant. When the ant needs to drop off the loaded object, all the locations recorded in the space can be first examined, and the potential clustering location can then be examined; thus, the blindness of direct global search can be reduced, thereby lowering the clustering time and system expenses.

### 4.3 Acquisition of typical process route

When the clustering output results have been obtained from the method mentioned in Section 4.2, the most representative machining process route in each cluster of the technical process can be extracted as the typical process route. It is the most representative process case in its technical process, which determines that the mean similarity is inevitably the highest compared with other process routes in the same cluster. Therefore, the typical process route can be obtained by taking the maximum mean similarity among all data objects and others in each cluster. The mean similarity formula for any process route *M* in some cluster to the given cluster can be expressed as

$$ASIM(M)=\frac{1}{|cluster|-1}\sum_{N\in |cluster|,\;M\neq N}SIM(M,N),$$ (8)

where *ASIM*(*M*) refers to the mean similarity value of *M* relative to other elements in the cluster. *M* is contained in a cluster, and |*cluster*| denotes the number of elements in the cluster. When the mean similarity values of all elements in the cluster are calculated, the corresponding process route with the maximum value can be considered as the typical process route.

## 5. Discussion of reuse approaches for the typical process route

### 5.1 Direct revision of typical process route

For some series products, their design objective is relatively similar to the typical process case; under such circumstances, some procedures or parameters of the typical process route can be revised to generate the new machining plan.

The typical process route library has been established by mining the corporate process cases. Meanwhile, the parameterisation of data in the technical process of the typical process route was carried out. The design with an identical technical process can be directly realised by modifying the parameters in the typical process route. Furthermore, such a route can be considered as a typical case of some type of design object, which can serve as reference for applying the CBR technique while designing the new process. The new process plan can be generated by extracting, screening, and modifying the process information.

### 5.2 Indirect matching of typical process route

The technical process design of parts is extremely complicated, which can be affected by many factors, including constraints of manufacturing requirements and selection of manufacturing resources. The core link of such design lies in the planning of the process route, which can also be considered as a problem of machining operation selection and procedure ordering constraint. Regarding the procedure ordering constraint, the predefined constraint condition and sorting objective function should be adopted to provide a reference for the organisation and arrangement of machining operation sequence; thus, the final output process route sequence scheme can be guaranteed to approximate or satisfy the optimal solution. During the process, it is generally required to set up the constraint condition of machining operation sequence and sorting objective function; in addition, the intelligence optimisation algorithm would be employed to solve the decision-making of the process route.

The sorting constraint conditions mainly include the process and manufacturing resource constraints. The former mainly refers to some technical process design criteria that should be complied with during the arrangement of the process sequence, for example, ‘the rough machining should precede the fine machining’, ‘the benchmark shall proceed firstly’, and ‘the surface should precede the holes’. The latter mainly refers to selecting and replacing manufacturing resources to arrange the process sequence. In production practice, it is inevitably time-consuming if the generated process route frequently needs to change machine tools or requires multi-time clamping and positioning. Furthermore, a large accumulative error may occur, increasing the production cost and lowering the machining quality of parts. Therefore, it is essential to establish objective functions according to sorting constraint conditions. Subsequently, the objective function can be utilised to evaluate various sorting schemes of the process route. The principle to formulate the sorting objective function is to ensure the minimum turnover rate of manufacturing resources based on the satisfaction of process constraints.

The mechanism to assist the decision-making of process routes with an intelligence optimisation algorithm is to continuously generate new machining process route sorting schemes through the iterative loop of the mentioned algorithm. Subsequently, the pre-established objective function is utilised to evaluate the sorting schemes. The current optimal solution would be replaced if the more superior sorting scheme appears. Otherwise, the iterative loop would continue. If the number of iterative loops of the optimisation algorithm reaches the set value or the continuous iterative loops reaches the limit without a more superior solution, the algorithm would stop operating; then, the optimal solution of the output end state is the final sorting scheme.

It is not the research focus of this study to utilise the iterative loop of intelligence optimisation algorithm to continuously generate the new sorting scheme of the process routes. The reuse concept of indirect matching of the typical process route proposed herein applies the typical process route for the category to which the design objective belongs in order to establish the sorting objective function, thus achieving the purpose of considering the typical process route as a reference. The arrangement of procedure sequence for the typical process route contains the rich experience in process design. It can even represent the procedure position arrangement of machining scheme for similar parts to some extent. The similarity of the new scheme and typical process roue can be calculated when generating the new procedure sequence scheme through the continuous iterative loop of intelligence optimisation algorithm; the objective function with high evaluation value and similarity can thus be established. The merit of such deed is the omission of analysis and discussion of the sorting constraint conditions, enabling the reduction of construction difficulty for the decision optimisation model of the process route. As regards the calculation method of the typical process route, a detailed explanation is provided in Section 4.

## 6. Case study

### 6.1 Similarity calculation of machining process routes

The process instances of some company’s products are adopted for verification. Ten process routes, including parts of axles, sleeves, flange plates and caps, and boxes, are randomly selected from the company’s instances library. The machining process path of these 10 process routes is extracted, as shown in Table 2.

**Table 2:** The process routes extracted from instances.

Part number	Part name	Machining process route
1	drive shaft	rough turning→semi-finish turning→finish turning→rough milling →semi-finish milling→finish milling→grinding
2	stepped shaft	rough turning→semi-finish turning→finish turning→milling
3	cylinder liner	casting→rough turning→semi-finish turning→boring
4	guide sleeve	rough turning→semi-finish turning→drilling→boring→grinding
5	bearing Cover	rough turning→semi-finish turning→drilling→turning (inner circle) →milling→drilling
6	flange	casting→rough turning→semi-finish turning→finish turning→drilling →rough turning(inner circle)→finish turning(inner circle) →drilling
7	valve cover	casting→rough turning→semi-finish turning→finish turning →boring(centre hole)→drilling
8	close lid	casting→rough turning→semi-finish turning→milling→drilling(reaming)
9	Triangular bearing box	casting→milling→milling→rough boring→finis boring→milling →drilling→milling→drilling
10	valve body	casting→milling→milling→milling→rough boring→finish boring →rough boring→drilling(tapping)→drilling(tapping)

As detailed in Section 3, the similarity values for these 10 cases are calculated (the computational code of similarity calculation case in S1 File), then a matrix of similarity values **SIM**_10×10_ can be constructed as follows:

$$\mathrm{SIM}=[\begin{array}{cccccccccc} 1.0000 & 0.5714 & 0.2857 & 0.4286 & 0.4286 & 0.3750 & 0.4286 & 0.4286 & 0.0582 & 0.0546\\ & 1.0000 & 0.5000 & 0.4000 & 0.5000 & 0.3750 & 0.5000 & 0.6000 & 0.0582 & 0.0546\\ & & 1.0000 & 0.6000 & 0.3333 & 0.3750 & 0.6667 & 0.6000 & 0.2222 & 0.1837\\ & & & 1.0000 & 0.4167 & 0.3220 & 0.5000 & 0.6000 & 0.0798 & 0.0726\\ & & & & 1.0000 & 0.6116 & 0.4167 & 0.5833 & 0.2542 & 0.0546\\ & & & & & 1.0000 & 0.5720 & 0.4470 & 0.3122 & 0.1111\\ & & & & & & 1.0000 & 0.6667 & 0.3020 & 0.1837\\ & & & & & & & 1.0000 & 0.2491 & 0.1657\\ & & & & & & & & 1.0000 & 0.6896\\ & & & & & & & & & 1.0000 \end{array}].$$

**Fig 3:**
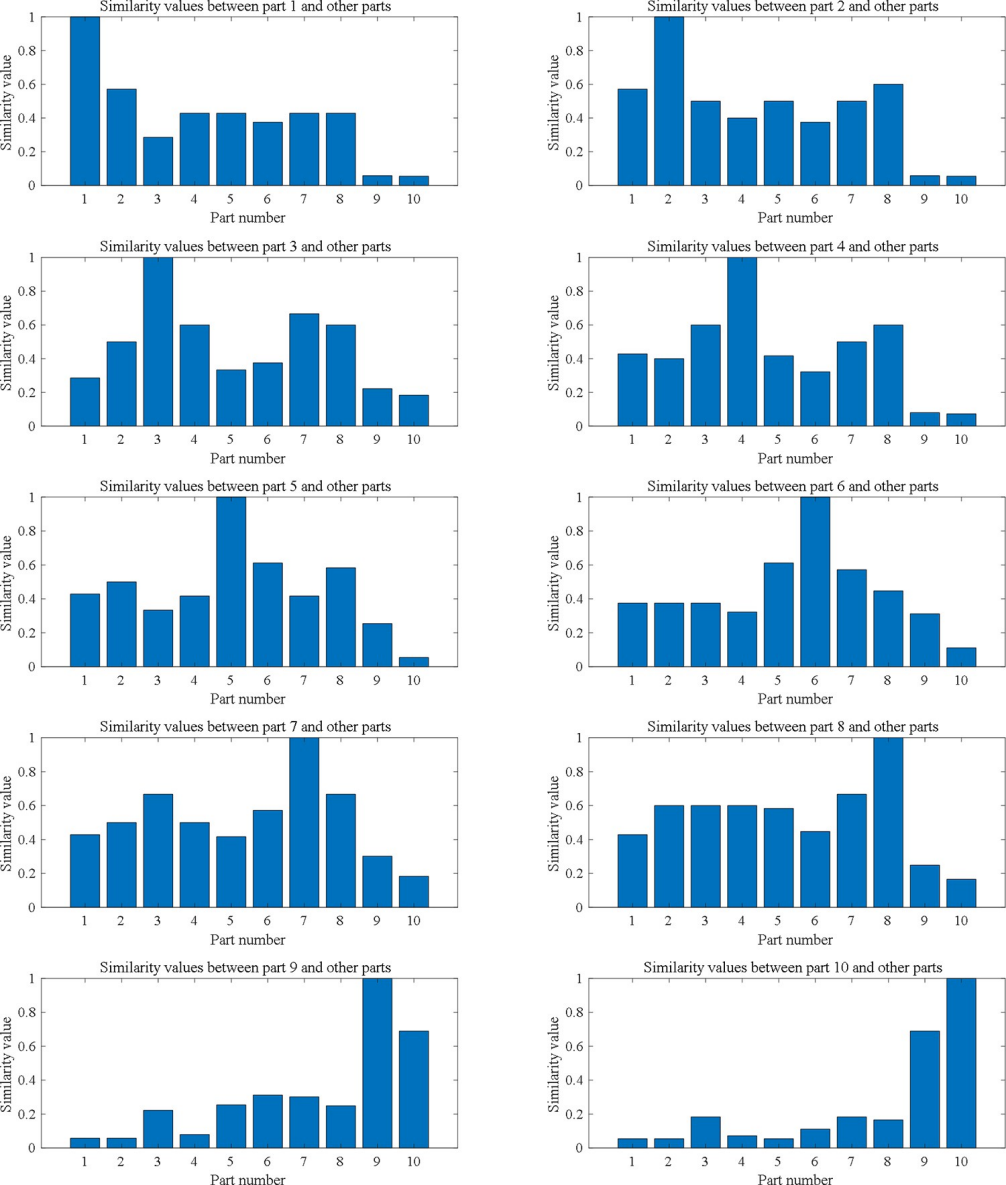
Similarity calculation results of process routes.

Take part 1 and 2 as an example, the value of matrix element **SIM**(1, 2) can be used to represent their process route similarity. It is obvious that **SIM** is a symmetric matrix. For each part, in order to make its route similarity with other parts be more intuitive and clear, ten histograms are used to demonstrate as shown in Fig 3. Evidently, except for the complete identity of cases themselves, the similarity of the technical process of essentially similar parts are clearly greater than that of the parts are not in the same type. For example, part 1 and 2 are axles, part 3 and 4 are sleeves, and part 9 and 10 are boxes. They have considerable similarities in their process routes, consistent with the actual manufacturing process and experience recognition.

### 6.2 Discovery of typical process route

Clustering of 10 process routes shown in Table 2 is considered as an example for demonstration. As the number of data objects to be clustered *Num* = 10, according to the description in Section 4.2, the number of iterative loops can be taken as *Gen* = 100 and the number of ants *N*_*ant*_ = *Num*/5 = 2. The 2D grid plane is initialised and takes the value range of horizontal and vertical coordinates as $X=Y=3\sqrt{Num}\approx 10$. The number of data objects to be clustered is 10, which is a small-scale clustering problem. Therefore, the threshold of swarm similarity can be selected to determine whether the ant should pick up or drop off the process route. The symbolic entropy-based similarity measurement method proposed herein integrates the global and local similarity of the process route. Therefore, the judgement standard of similarity is stricter. As a result, the decision threshold of swarm similarity can be set as 0.5.

**Fig 4:**
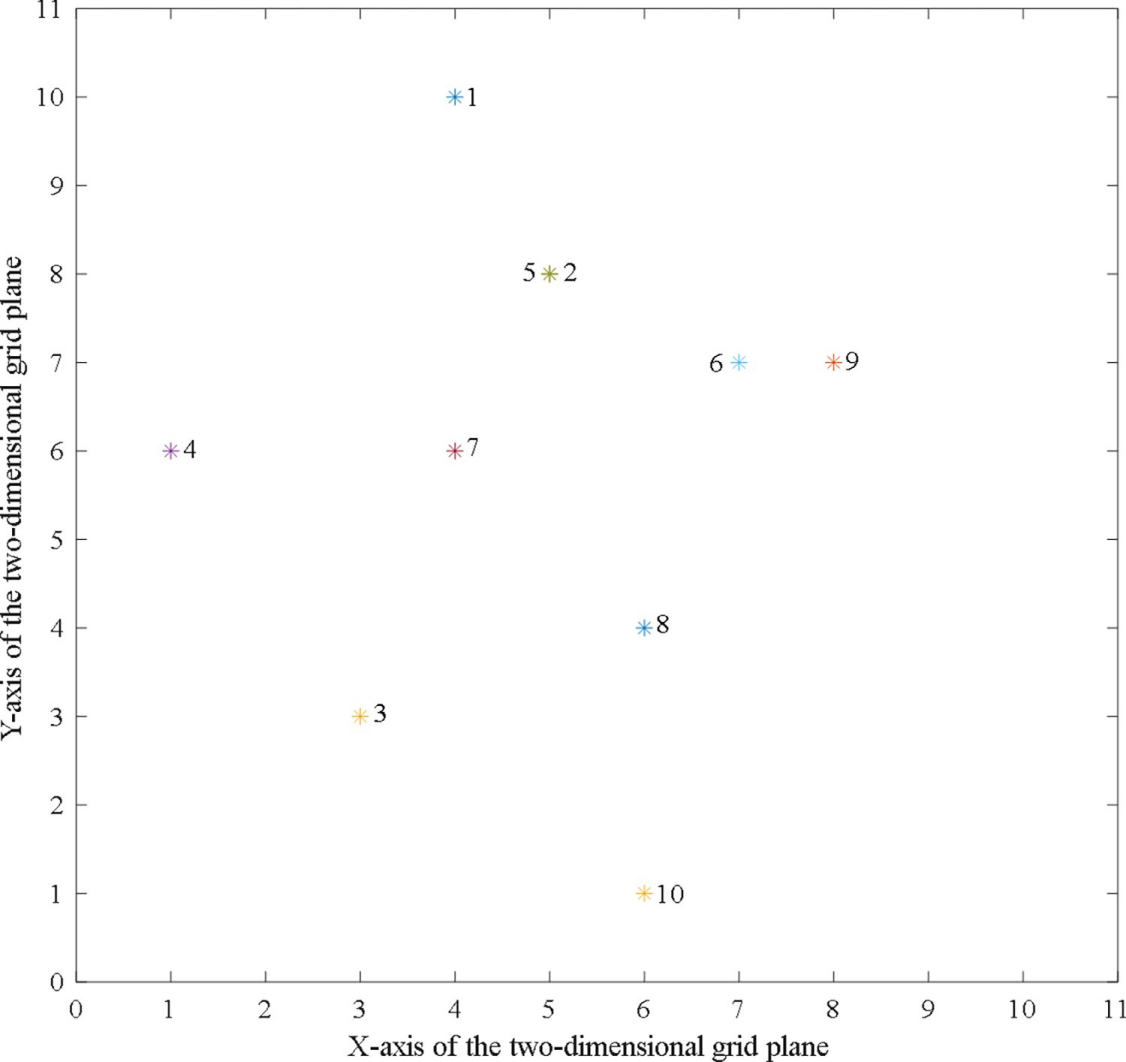
The initial location distribution of process routes to be clustered.

The aforementioned parameters are input into the program model according to the clustering steps described in Section 4.2. Subsequently, the initial distribution of the process route shown in Fig 4 can be obtained. The symbol “*” in the figure indicates the location of the process route.

**Fig 5:**
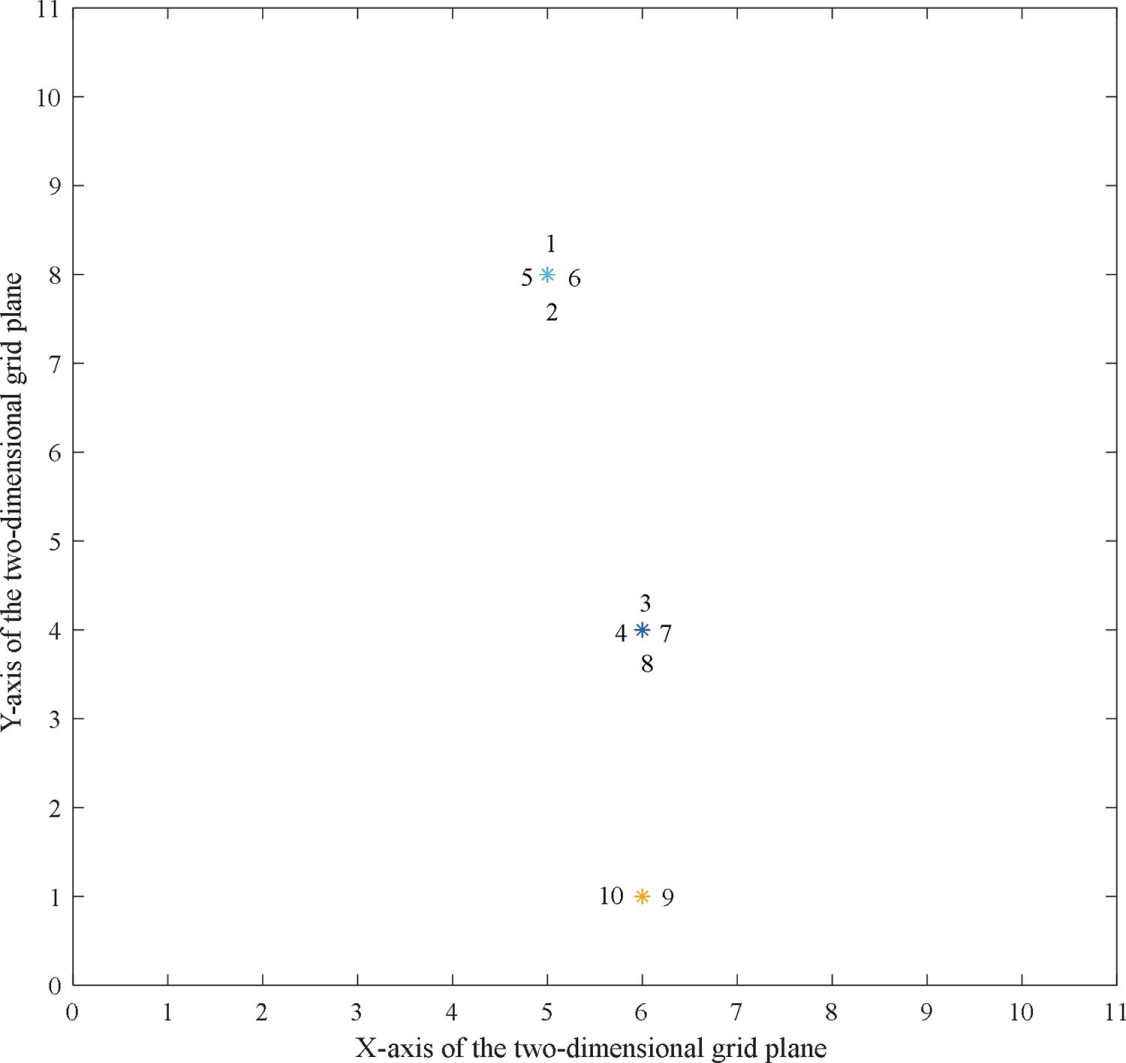
The clustering partition results.

Subsequently, the clustering partition results shown in Fig 5 can be obtained by running the clustering program (the computational code of clustering analysis case in S2 File).

As indicated by Fig 5, the clustering partition results are as follows: *cluster*_1_ = {part 1, part 2, part 5, part 6}, *cluster*_2_ = {part 3, part 4, part 7, part 8}, and *cluster*_3_ = {part 9, part 10}. Thus, axles, sleeves, and boxes are still respectively categorised. Owing to the great difference of process, the plates and caps are categorised respectively into *cluster*_1_ and *cluster*_2_ based on the principle of a similar process.

**Fig 6:**
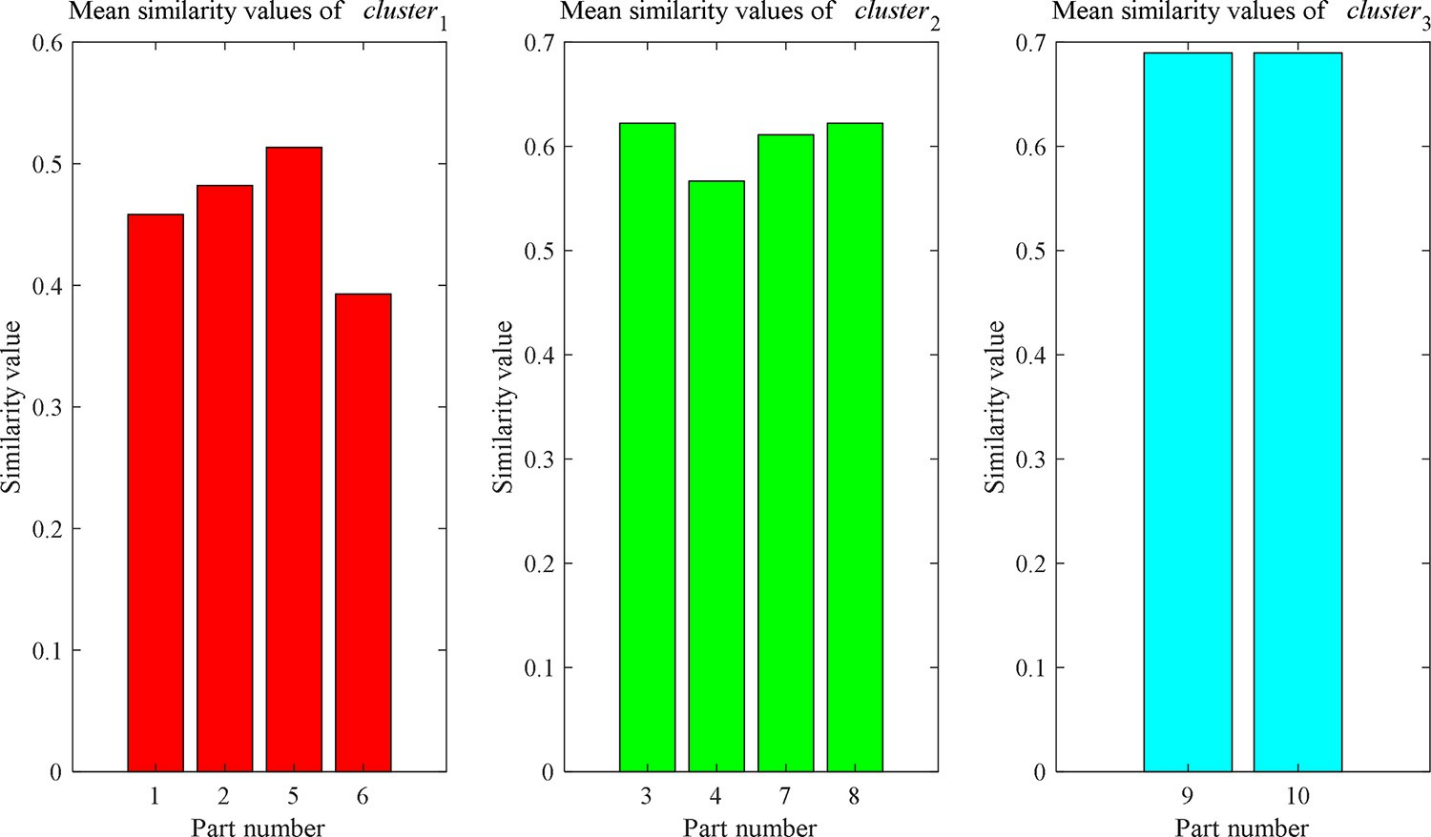
The mean similarity calculation results of each category.

According to the clustering partition results, the mean similarity for each part in the corresponding class can be calculate by Eq (8), which are shown in Fig 6. Evidently, the typical process route for *cluster*_1_ is part 5; part 3 or part 8 is the typical process route for *cluster*_2_; and *cluster*_3_ corresponds to part 9 or part 10.

### 6.3 Process reuse based on typical process route

Two reuse approaches of the typical processes are introduced herein. The technologists can adopt direct revision to generate new processes through analysis, screening, and revision based on the typical process route. Such an approach emphasises the technologist’s subjective initiative, and the implementation course is relatively simple. Therefore, further explanation is not provided herein. The cases of indirect matching of typical process routes are considered for further discussion.

**Fig 7:**
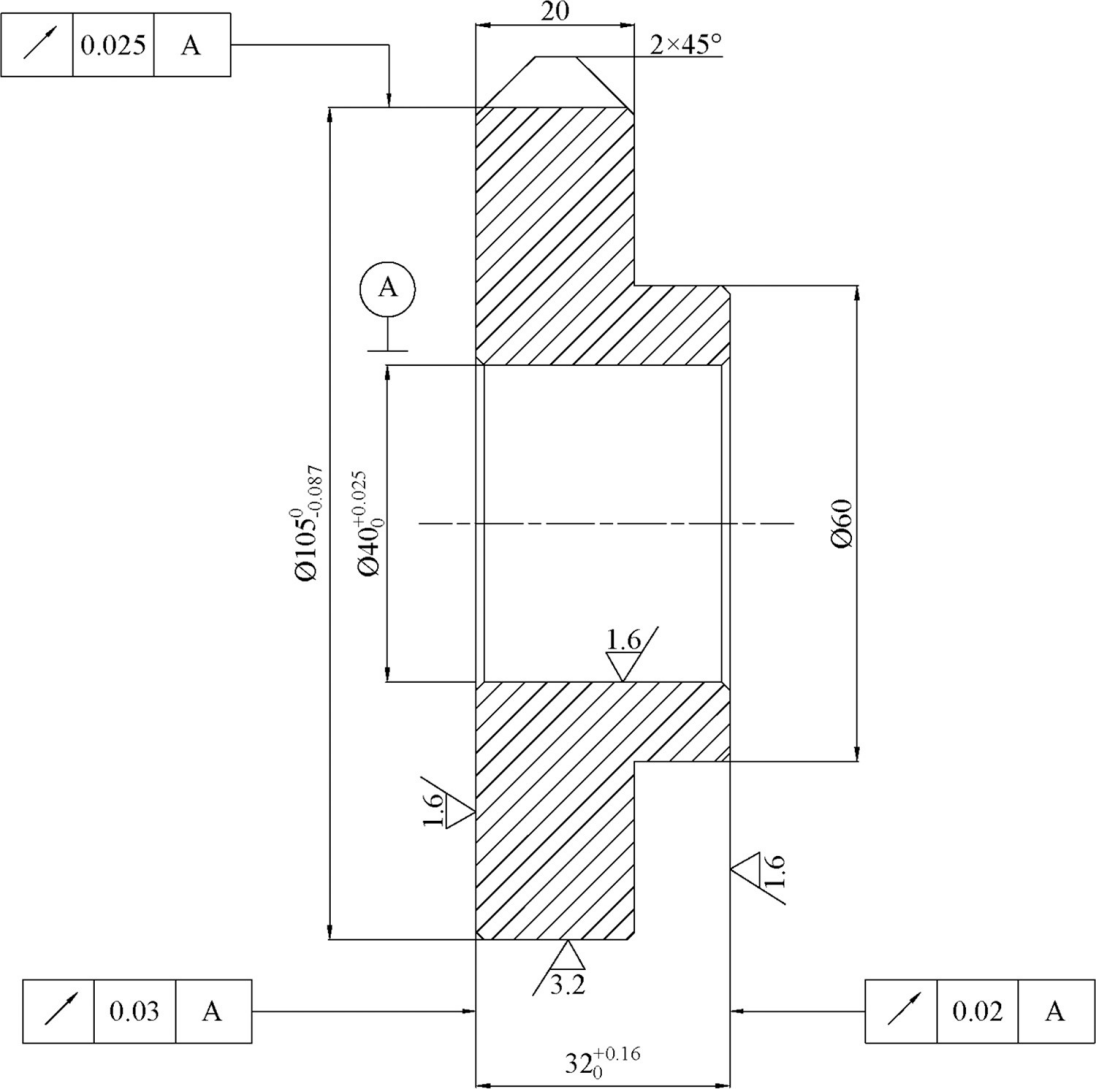
Example part.

**Fig 8:**
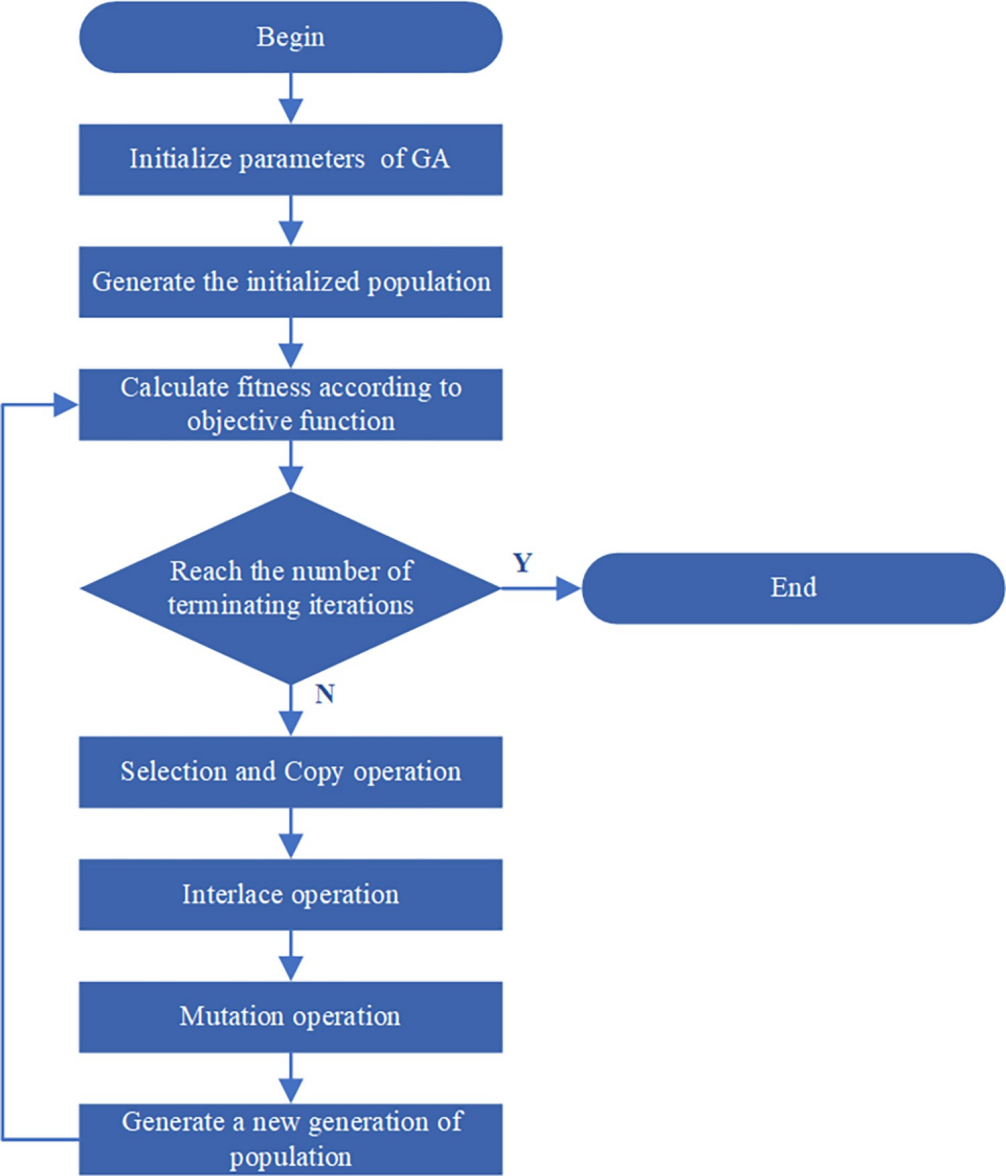
The realisation process of genetic algorithm.

**Fig 9:**
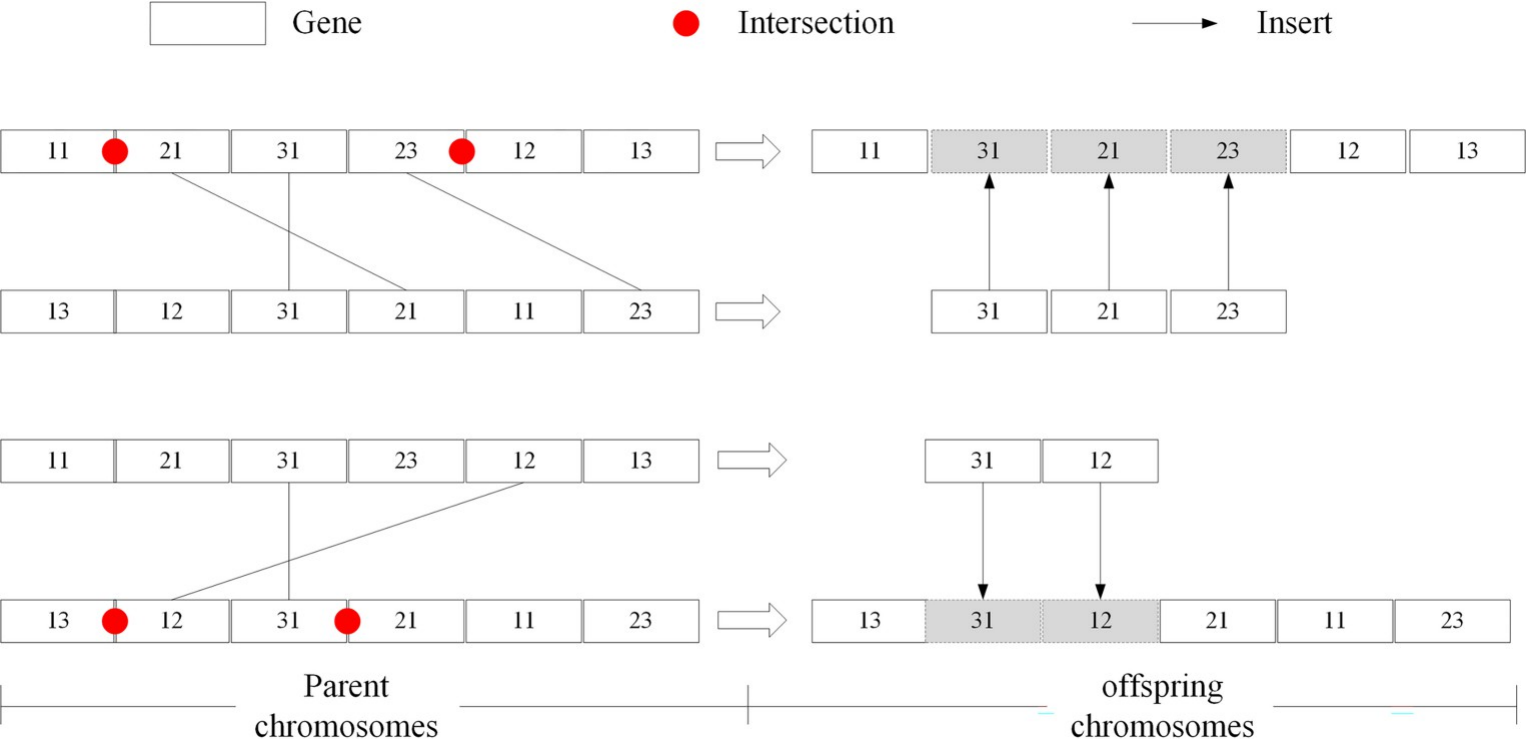
Interlace operation process of chromosome.

**Fig 10:**
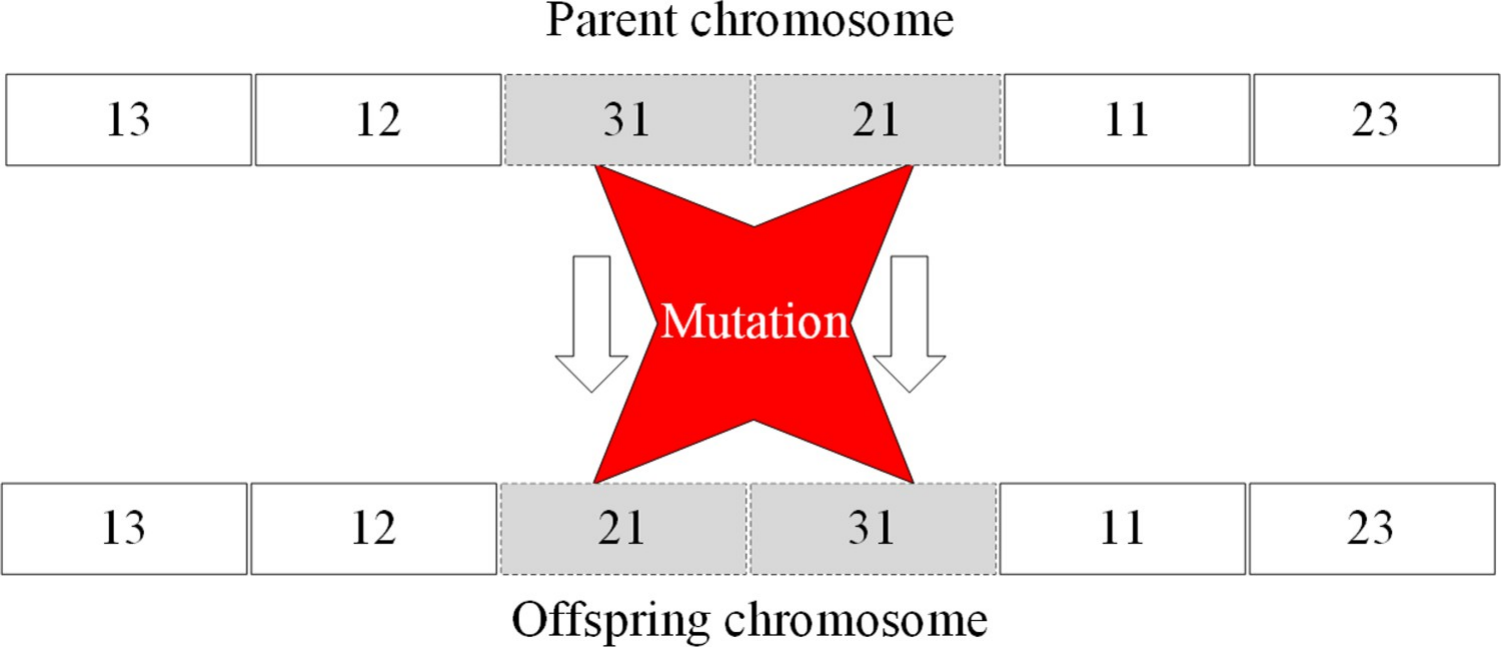
Mutation operation process of chromosome.

The formulation of a machining scheme based on the matching level of the typical process route is to apply the intelligence optimisation algorithm to the decision course of the process route. Consider the genetic algorithm as an example. It repeatedly applies genetic operations to the population in the solution space to generate a new population, allowing the continuous optimisation of process route chromosome contained in each generation of the population. For example, a cover part shown in Fig 7 is taken to demonstrate the indirect matching of typical process route. The part mainly contains five machining features: the left and right end face, excircle with a diameter of 105, excircle with a diameter of 60 and a Ø40 centre hole. According to the process analysis of this example part, the machining method for the left end face is rough turning, and the method for the right end face is rough turning → finish turning; regarding Ø105 excircle, the machining method is rough turning → semi-finished turning → finish turning; as for Ø60 excircle, the method is rough turning → semi-finished turning; regarding the Ø40 centre hole, it involves in drilling → rough turning (inner circle) → finish turning (inner circle). The indirect reuse process of this example part based on GA mainly contains the following six steps (see Fig 8 for the realisation process):

1. Gene coding
  In this case, the chromosome stands for the potential process route of the part, and the gene signifies the procedure operation. According to the combination of manufacturing procedures, the number of the part’s procedures to be sorted for the part is six: rough turning, semi-finished turning, finish turning, drilling, rough turning (inner circle), and finish turning (inner circle). Evidently, a chromosome consists of six genes in total. When coding the genes, the simplest approach is to adopt the ‘machining method + machining stage’ method. For instance, the machining method of turning the excircle can be coded as 1; turning the inner circle as 2; and drilling as 3. Similarly, the code for the rough machining stage is 1, 2 indicates the semi-finished machining stage, and 3 refers to the finish machining stage. The coding without machining stage division can be coded according to the rough machining mode. The six procedures for the example part can be coded as 11, 12, 13, 31, 21, and 23.
2. Initialisation of population
  The length of the chromosome can determine the scale of the population, and the initialisation can proceed accordingly; thus, the solution space of the process route for the example part can be formed.
3. Determination of objective function
  The analysis indicates that the structure of the part is more approximate to the part in *cluster*_1_ obtained in Section 6.2. Therefore, the similarity between the typical process route of *cluster*_1_, namely, part 5, and the chromosome in the solution space would be selected to establish the objective function. The more similar the chromosome in the solution space to part 5’s process route, the more superior would be the solution. In Section 3, the solution to the similarity of two process routes through symbolic entropy is detailed; here, the same code is regarded as the same symbol when calculating similarity. Based on the similarity, the objective function *F* is constructed as
$$F=\min(\frac{1}{SIM(L,L^{\prime })}),$$ (9)
  where *L* stands for the typical process route, which specifically refers to the route of part 5. The coded process route is 11→12→31→21→41→31; *L*′ signifies the represented process route of chromosome in the solution space.
4. Copy operation
  The copy probability should be set, and the superior chromosome in the parent population should be duplicated to the next generation population based on the probability.
5. Interlace operation
  Firstly, two chromosomes is randomly selected as the parent chromosomes, and two crossover locations is randomly generated in each parent chromosome. Subsequently, the gene between the intersection of one of the parent chromosomes is picked up; such genes are re-sorted according to their corresponding sequence in the other parent chromosome. Finally, they are inserted between two intersections to generate a new chromosome. The principle of interlace operation is depicted in Fig 9.
6. Mutation operation
  Mutation operation can be understood as the internal interlace operation in the chromosome. Some chromosomes can be selected according to the preset mutation rate to directly exchange the positions of two genes internally, thus forming the new chromosome. The principle of mutation operation is shown in Fig 10.

Genetic algorithm as a intelligent optimization algorithm has lots of reports in various scholarly literature, and there are some validated and effective value ranges for its parameters. After analyzing these applications from literature, I found that the usual range of chromosome population is from 10 to 100, the usual range of copy probability is from 10% to 20%, the usual range of interlace probability is from 50% to 99%, the usual range of mutation rate is from 0.01% to 10%. On this basis, in consideration of the example part shown in Fig 7 only containing six procedures to be sorted, and a number of parameters selection trials were carried out, the relative parameters are identified as follows: the scale of chromosome population can be taken as 10, copy probability is 10%, interlace probability is 70%, the mutation rate is 5%, and the number of genetic cycle iterations is 100.

**Fig 11:**
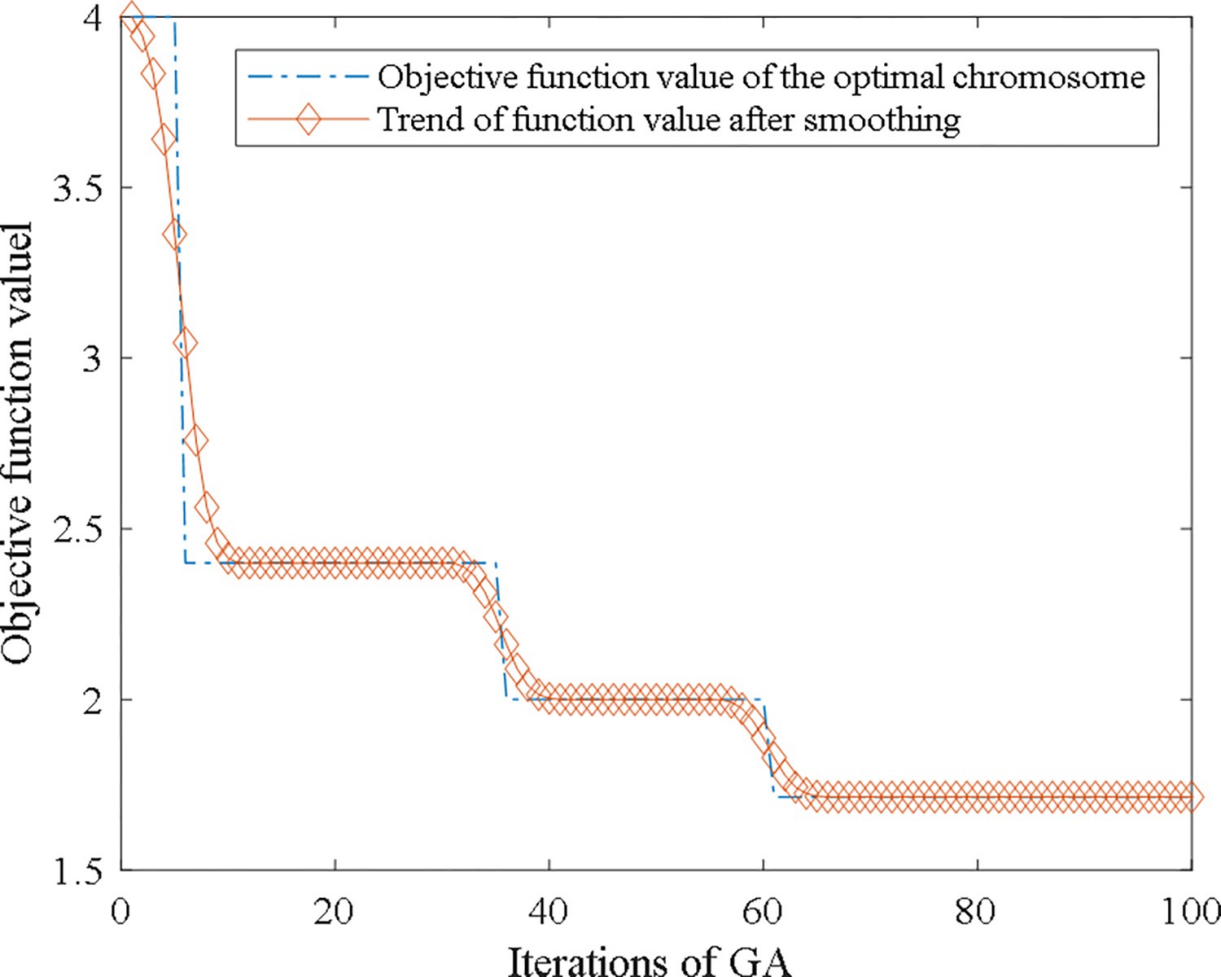
The convergence curve of the objective function.

**Fig 12:**
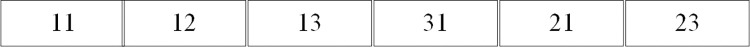
Optimal chromosome.

The genetic sequencing process of the process route can be programmed according to the aforementioned steps. The convergence curve of algorithm operation is shown in Fig 11. Process design criteria are used to screen for the optimal chromosomes searched from each iteration, and the optimal chromosome found in the search is shown in Fig 12. The process route described by Fig 12 can be expressed as follows: rough turning → semi-finished turning → finish turning → drilling → rough turning (inner circle) → finish turning (inner circle). In the solution space, the finally obtained process route has higher similarity with the typical process route of the class cluster where the machine part shown in Fig 7 belongs to, thus representing its procedure position arrangement meet some common requirements for process design in this kind of parts, and illustrating it is potentially reasonable. In addition, this result is also consistent with the cognition of machining designer, which verifies correctness of the searched optimal chromosome.

## 7. Conclusions

This study entailed the development of a novel framework for discovering and reusing a typical process route. The proposed method has a clear target that the reuse efficiency of manufacturing instances must be improved. In this framework, similarity analysis, mining, and reuse of manufacturing instances implemented different degrees of innovation, raising the accuracy of the reuse object’s determination, and expanding the reuse approach. In addition, the proposed framework comprehensively covers the aspects of similarity analysis, mining, and reuse of manufacturing instances; thus, it can provide better support for process instance reuse.

The poor effectiveness of the auto-generated machining process route is one of the restricting factors to the development of intelligent manufacturing. Therefore, the proposed framework is a good choice for process route planning in intelligent manufacturing and is also closer to the research focus and emerging research direction in the knowledge engineering field. The research is still in its initial stages; however, the applied case demonstrates that the proposed method is feasible and effective. On the basis of this framework, the future research direction will be optimising and improving manufacturing instance mining and reuse system, such as establishing a multi-dimensional similarity measurement model or realising intelligent reuse driven by the designer’s subjective intent.

## Supporting information

S1 File The computational code of similarity calculation case in S1 File. (DOCX)

S2 File The computational code of clustering analysis case in S2 File. (DOCX)
