# Supplementary material for: A novel framework for discovery and reuse of typical process route driven by symbolic entropy and intelligent optimisation algorithm
Source: PLoS One. 2022 Sep 12;17(9):e0274532. doi: 10.1371/journal.pone.0274532 (PMC9467352; doi:10.1371/journal.pone.0274532)
Supplement: S1 File — (DOCX) [file pone.0274532.s001.docx]

% ..................................................................MAIN

clear;

M{1}='ABCDEFM';

M{2}='ABCD';

M{3}='HABT';

M{4}='ABZTM';

M{5}='ABZUDZ';

M{6}='HABCZUVZ';

M{7}='HABCTZ';

M{8}='HABDZ';

M{9}='HDDTRDZDZ';

M{10}='HDDDTRTDD';

SIMM=zeros(10,10);

for i=1:9

for j=(i+1):10

SIMM(i,j)=smstr(M{i},M{j});

end

end

SIMM=SIMM+SIMM';

for i=1:10

SIMM(i,i)=1;

end

figure(1)

subplot(5,2,1)

y =SIMM(1,:);

bar(y);

Cell = {'1', '2', '3','4', '5', '6','7', '8', '9','10'};

set(gca, 'XTick',1:10, 'XTickLabel',Cell);

ylabel('Similarity value')

% ylabel('Average Throughout (per Second)')

xlabel('Part number')

title('Similarity values between part 1 and other parts')

subplot(5,2,2)

y =SIMM(2,:);

bar(y);

Cell = {'1', '2', '3','4', '5', '6','7', '8', '9','10'};

set(gca, 'XTick',1:10, 'XTickLabel',Cell);

ylabel('Similarity value')

% ylabel('Average Throughout (per Second)')

xlabel('Part number')

title('Similarity values between part 2 and other parts')

subplot(5,2,3)

y =SIMM(3,:);

bar(y);

Cell = {'1', '2', '3','4', '5', '6','7', '8', '9','10'};

set(gca, 'XTick',1:10, 'XTickLabel',Cell);

ylabel('Similarity value')

% ylabel('Average Throughout (per Second)')

xlabel('Part number')

title('Similarity values between part 3 and other parts')

subplot(5,2,4)

y =SIMM(4,:);

bar(y);

Cell = {'1', '2', '3','4', '5', '6','7', '8', '9','10'};

set(gca, 'XTick',1:10, 'XTickLabel',Cell);

ylabel('Similarity value')

% ylabel('Average Throughout (per Second)')

xlabel('Part number')

title('Similarity values between part 4 and other parts')

subplot(5,2,5)

y =SIMM(5,:);

bar(y);

Cell = {'1', '2', '3','4', '5', '6','7', '8', '9','10'};

set(gca, 'XTick',1:10, 'XTickLabel',Cell);

ylabel('Similarity value')

% ylabel('Average Throughout (per Second)')

xlabel('Part number')

title('Similarity values between part 5 and other parts')

subplot(5,2,6)

y =SIMM(6,:);

bar(y);

Cell = {'1', '2', '3','4', '5', '6','7', '8', '9','10'};

set(gca, 'XTick',1:10, 'XTickLabel',Cell);

ylabel('Similarity value')

% ylabel('Average Throughout (per Second)')

xlabel('Part number')

title('Similarity values between part 6 and other parts')

subplot(5,2,7)

y =SIMM(7,:);

bar(y);

Cell = {'1', '2', '3','4', '5', '6','7', '8', '9','10'};

set(gca, 'XTick',1:10, 'XTickLabel',Cell);

ylabel('Similarity value')

% ylabel('Average Throughout (per Second)')

xlabel('Part number') %

title('Similarity values between part 7 and other parts')

subplot(5,2,8)

y =SIMM(8,:);

bar(y);

Cell = {'1', '2', '3','4', '5', '6','7', '8', '9','10'};

set(gca, 'XTick',1:10, 'XTickLabel',Cell);

ylabel('Similarity value')

% ylabel('Average Throughout (per Second)')

xlabel('Part number')

title('Similarity values between part 8 and other parts')

subplot(5,2,9)

y =SIMM(9,:);

bar(y);

Cell = {'1', '2', '3','4', '5', '6','7', '8', '9','10'};

set(gca, 'XTick',1:10, 'XTickLabel',Cell);

ylabel('Similarity value')

% ylabel('Average Throughout (per Second)')

title('Similarity values between part 9 and other parts')

subplot(5,2,10)

y =SIMM(10,:);

bar(y);

Cell = {'1', '2', '3','4', '5', '6','7', '8', '9','10'};

set(gca, 'XTick',1:10, 'XTickLabel',Cell);

ylabel('Similarity value')

% ylabel('Average Throughout (per Second)')

xlabel('Part number')

title('Similarity values between part 10 and other parts')

% ..................................................................smstr

function sm=smstr(sa,sb)

[cstr,lenn,A,B]= lcs(sa,sb,1)

ai=[];

qi=[];

hi=[];

for i=1:lenn

idx=strfind(sa,cstr(i));

aa=0;

a=[];

for j=1:length(idx)

a(j)=1/(idx(j)-aa);

aa=idx(j);

end

[m]=find(idx==A(i));

if m==1

ai(i)=(1/idx(m));

qi(i)=(ai(i)/sum(a));

hi(i)=(-qi(i)*log2(qi(i)));

else

ai(i)=(1/(idx(m)-idx(m-1)));

qi(i)=(ai(i)/sum(a));

hi(i)=(-qi(i)*log2(qi(i)));

end

end

ai2=[];

qi2=[];

hi2=[];

for i=1:lenn

idx=strfind(sb,cstr(i));

aa=0;

a=[];

for j=1:length(idx)

a(j)=1/(idx(j)-aa);

aa=idx(j);

end

[m]=find(idx==B(i));

if m==1

ai2(i)=(1/idx(m));

qi2(i)=(ai2(i)/sum(a));

hi2(i)=(-qi2(i)*log2(qi2(i)));

else

ai2(i)=(1/(idx(m)-idx(m-1)));

qi2(i)=(ai2(i)/sum(a));

hi2(i)=(-qi2(i)*log2(qi2(i)));

end

end

q=0;

for i=1:lenn

q=(q+abs(hi(i)-hi2(i)));

end

sm=((lenn/max(length(sa),length(sb)))*(1-(1/lenn)*q));

% ..................................................................lcs

function [cstr,lenn,A,B]= lcs(sa,sb,mode)

%LCS Longest common substring or subsequence

% LCS(A,B) returns the longest common substring of vectors A v.s. B,

% which means if there exists two or more substrings, just the first

% substring in A is returned.

%

% LCS(A,B,MODE) returns the longest common subsequence of vectors A v.s.

% B for mode is TRUE.

%

% The longest common substring is the longest string (or strings) that

% is a substring (or are substrings) of two or more strings. However,

% the longest common subsequence (LCS) is the longest subsequence common

% to two (or more) sequences.

%

% Examples:

% % Find the substring sa v.s. sb:

% sa=[2 1 2 3 2 5 2 3 3 1 1 3 2 4];

% sb=[3 1 2 1 2 3 2 2 3 4 4 5];

% lcs(sa,sb) % returns [2 1 2 3 2]

%

% % Get the subsequence sa v.s. sb:

% sa='ACGTAACCT'; sb='GGACTAGG';

% lcs(sa,sb,1) % returns 'ACTA'

% % sb v.s. sa:

% lcs(sb,sa,1) % returns 'GACT'

%

% See also: STRFIND.

% $Author: kastin $

% $Revision: 1.0 $ $Date: 2013/07/05 16:00:08 $

sa1=sa;

sb1=sb;

A=[];

B=[];

isseq=false; % substring

cstr=[];

if nargin<2

error('MATLAB:lcs','Not enough input arguments.');

elseif nargin==3

isseq=mode;

end

if isempty(sa) || isempty(sb), return; end

lena=length(sa);

lenb=length(sb);

lena1=length(sa1);

lenb1=length(sb1);

c = zeros(size(sb));

if ~isseq

endp=0;

len=0;

for i=1:lena

for j=lenb:-1:1

if sa(i) == sb(j)

if(i==1||j==1)

c(j)=1;

else

c(j)=c(j-1)+1;

end

else

c(j) = 0;

end

if c(j) > len

len=c(j);

endp=j;

end

end

end

start=endp-len+1;

cstr=sb(start:endp);

else

r=zeros(lena+1,lenb+1);

d=zeros(lena+1,lenb+1);

for i=2:lena+1

for j=2:lenb+1

if sa(i-1) == sb(j-1)

r(i,j)=1;

d(i,j)=d(i-1,j-1)+1;

else

if d(i-1,j)>=d(i,j-1)

d(i,j) = d(i-1,j);

r(i,j)=2;

else

d(i,j) = d(i,j-1);

r(i,j)=3;

end

end

end

end

idx=[];

while i~= 1 && j~= 1

switch r(i,j)

case 1

idx=[i,idx];

i=i-1;j=j-1;

case 2

i=i-1;

case 3

j=j-1;

end

end

cstr=sa(idx-1);

end

lenn=length(cstr);

tt=1;

for i=1:lena1

if sa1(i)==cstr(tt)

A(tt)=i;

if tt<length(cstr)

tt=tt+1;

end

end

end

ttt=1;

for i=1:lenb1

if sb1(i)==cstr(ttt)

B(ttt)=i;

if ttt<length(cstr)

ttt=ttt+1;

end

end

end
