# Supplementary material for: A novel framework for discovery and reuse of typical process route driven by symbolic entropy and intelligent optimisation algorithm
Source: PLoS One. 2022 Sep 12;17(9):e0274532. doi: 10.1371/journal.pone.0274532 (PMC9467352; doi:10.1371/journal.pone.0274532)
Supplement: S2 File — (DOCX) [file pone.0274532.s002.docx]

clear;

M{1}='ABCDEFM';

M{2}='ABCD';

M{3}='HABT';

M{4}='ABZTM';

M{5}='ABZUDZ';

M{6}='HABCZUVZ';

M{7}='HABCTZ';

M{8}='HABDZ';

M{9}='HDDTRDZDZ';

M{10}='HDDDTRTDD';

SIMM=zeros(10,10);

for i=1:9

for j=(i+1):10

SIMM(i,j)=smstr(M{i},M{j});

end

end

SIMM=SIMM+SIMM';

for i=1:10

SIMM(i,i)=1;

end

A=SIMM;

Num=size(A,1);

X=ceil(3*Num^0.5);

Y=ceil(3*Num^0.5);

OLID=zeros(Num,2);

OLcon=zeros(Num,1);

%m=Num/5;

m=2;

mID=zeros(m,2);

mcon=zeros(m,1);

mpick_OL=zeros(Num,1);

for i=1:2*m %定义放下位置记忆体

mdrop_postion{i}=[];

end

Gen=1000;

%R=2;

%R=1.5;

Nmin=2;

OL_m=zeros(m,1);%指定的对应数据对象

for i=1:m %指定过的记录

m_OL{i}=zeros(Num,1);

end

%初始化工艺路线位置

for i=1:Num

x=unidrnd(X);

y=unidrnd(Y);

OLID(i,1)=x;

OLID(i,2)=y;

end

figure (1)

for i=1:Num

plot(OLID(i,1),OLID(i,2),'*');

hold on

end

for i=1:Num

text(OLID(i,1),OLID(i,2),num2str(i));

end

%给蚂蚁指定初始数据对象

for i=1:m

x=unidrnd(Num);

mID(i,1)=OLID(x,1);

mID(i,2)=OLID(x,2);

OL_m(i)=x;

m_OL{i}(OL_m(i))=1;

end

%聚类迭代

for i=1:Gen

for j=1:m

%-------------------------------

if mcon(j)==0

n=0;

sim=[];

for cc=1:Num

if ((OLID(cc,1)-mID(j,1))^2+(OLID(cc,2)-mID(j,2))^2)^0.5==0 && cc~=OL_m(j)

n=n+1;

sim(n)=A(cc,OL_m(j));

end

end

if n==0

mcon(j)=1;

OLcon(OL_m(j))=1;

end

if n>=1

%if P_pick(sum(sim)/length(sim))>=rand

if (sum(sim)/length(sim))<0.5

mcon(j)=1;

OLcon(OL_m(j))=1;

else

x=unidrnd(Num);

mID(j,1)=OLID(x,1);

mID(j,2)=OLID(x,2);

OL_m(j)=x;

m_OL{j}(OL_m(j))=1;

end

end

%--------------------------

else

if length(mdrop_postion{2*j})==0

x=unidrnd(Num);

mID(j,1)=OLID(x,1);

mID(j,2)=OLID(x,2);

OLID(OL_m(j),1)=OLID(x,1);

OLID(OL_m(j),2)=OLID(x,2);

n=0;

sim=[];

for cc=1:Num

if ((OLID(cc,1)-mID(j,1))^2+(OLID(cc,2)-mID(j,2))^2)^0.5==0 && cc~=OL_m(j)

n=n+1;

sim(n)=A(cc,OL_m(j));

end

end

if n>=1

%if P_drop(sum(sim)/length(sim))>=rand;

%if (sum(sim)/length(sim))>=0.7;

if (sum(sim)/length(sim))>=0.5;

mcon(j)=0;

OLcon(OL_m(j))=0;

mdrop_postion{2*j-1}(length(mdrop_postion{2*j-1})+1)=mID(j,1);

mdrop_postion{2*j}(length(mdrop_postion{2*j})+1)=mID(j,2);

x=unidrnd(Num);

mID(j,1)=OLID(x,1);

mID(j,2)=OLID(x,2);

OL_m(j)=x;

m_OL{j}(OL_m(j))=1;

end

end

end

if length(mdrop_postion{2*j})~=0

ttt=0;

for ccc=1:length(mdrop_postion{2*j})

OLID(OL_m(j),1)=mdrop_postion{2*j-1}(ccc);

OLID(OL_m(j),2)=mdrop_postion{2*j}(ccc);

mID(j,1)=mdrop_postion{2*j-1}(ccc);

mID(j,2)=mdrop_postion{2*j}(ccc);

n=0;

sim=[];

for cc=1:Num

if ((OLID(cc,1)-mID(j,1))^2+(OLID(cc,2)-mID(j,2))^2)^0.5==0 && cc~=OL_m(j)

n=n+1;

sim(n)=A(cc,OL_m(j));

end

end

if n>=1

%if P_drop(sum(sim)/length(sim))>=rand;

%if (sum(sim)/length(sim))>=0.7;

if (sum(sim)/length(sim))>=0.5;

mcon(j)=0;

OLcon(OL_m(j))=0;

mdrop_postion{2*j-1}(length(mdrop_postion{2*j-1})+1)=mID(j,1);

mdrop_postion{2*j}(length(mdrop_postion{2*j})+1)=mID(j,2);

x=unidrnd(Num);

mID(j,1)=OLID(x,1);

mID(j,2)=OLID(x,2);

OL_m(j)=x;

m_OL{j}(OL_m(j))=1;

ttt=1;

break;

end

end

end

if ttt==0

x=unidrnd(Num);

mID(j,1)=OLID(x,1);

mID(j,2)=OLID(x,2);

OLID(OL_m(j),1)=OLID(x,1);

OLID(OL_m(j),2)=OLID(x,2);

n=0;

sim=[];

for cc=1:Num

if ((OLID(cc,1)-mID(j,1))^2+(OLID(cc,2)-mID(j,2))^2)^0.5==0 && cc~=OL_m(j)

n=n+1;

sim(n)=A(cc,OL_m(j));

end

end

if n>=1

%if P_drop(sum(sim)/length(sim))>=rand;

%if (sum(sim)/length(sim))>=0.7;

if (sum(sim)/length(sim))>=0.5;

mcon(j)=0;

OLcon(OL_m(j))=0;

mdrop_postion{2*j-1}(length(mdrop_postion{2*j-1})+1)=mID(j,1);

mdrop_postion{2*j}(length(mdrop_postion{2*j})+1)=mID(j,2);

x=unidrnd(Num);

mID(j,1)=OLID(x,1);

mID(j,2)=OLID(x,2);

OL_m(j)=x;

m_OL{j}(OL_m(j))=1;

end

end

else

end

end

%--------------------------

end

end

end

figure (2)

for i=1:Num

plot(OLID(i,1),OLID(i,2),'*');

hold on

end

for i=1:Num

text(OLID(i,1),OLID(i,2),num2str(i));

end
